# Large uterine leiomyoma presenting as pseudo-Meigs’ syndrome with an elevated ca125: a case report and literature review

**DOI:** 10.1093/jscr/rjac253

**Published:** 2022-06-07

**Authors:** Mohamed Abdelgawad, Lutfi Barghuthi, Tyler Davis, Mahmoud Omar, Omar M Kamel, Jake Gibbons, Yury Ragoza, Hishaam Ismael

**Affiliations:** Department of Surgery, University of Texas Health Science Center, UT Health East Texas, Tyler, TX, USA; Department of Surgery, University of Texas Health Science Center, UT Health East Texas, Tyler, TX, USA; Department of Surgery, University of Texas Health Science Center, UT Health East Texas, Tyler, TX, USA; Department of Surgery, School of Medicine, Tulane University, New Orleans, LA, USA; Department of Surgery, School of Medicine, Tulane University, New Orleans, LA, USA; Department of Surgery, University of Texas Health Science Center, UT Health East Texas, Tyler, TX, USA; Department of Surgery, University of Texas Health Science Center, UT Health East Texas, Tyler, TX, USA; Department of Surgery, University of Texas Health Science Center, UT Health East Texas, Tyler, TX, USA

## Abstract

Ascites, pelvic mass and elevated CA-125 in females carry a grim prognosis, likely an ovarian carcinoma. However, more benign etiologies such as Meigs’ and pseudo-Meigs’ syndrome must be considered. Pseudo-Meigs’ syndrome presenting with an elevated CA-125 is rare and presents a diagnostic challenge. Medline and PubMed were queried for pseudo-Meigs’ syndrome cases. We present a 35-year-old female patient who presented with abdominal swelling and weight gain. Imaging demonstrated a 29-cm large intraabdominal mass with significant ascites with elevation of CA-125. Surgical resection was performed, and pathology identified uterine leiomyoma. Twenty-one cases of pseudo-Meigs’ syndrome were identified in the literature. Most patients presented with abdominal distention, and some also reported dyspnea. All patients, including our case, were treated surgically. No recurrence reported among these cases. Surgery is the mainstay for radical treatment in pseudo-Meigs’ syndrome. Resolution of the ascites and hydrothorax occurs following resection of the tumor.

## INTRODUCTION

Meigs’ syndrome was first classified in 1954 as a triad of benign ovarian fibroma or thecoma with both ascites and hydrothorax [1]. A clinically similar yet distinct pathological entity is pseudo-Meigs’ syndrome. Like Meigs’, pseudo-Meigs’ syndrome involves ascites and hydrothorax. However, it is associated with non-thecoma, non-fibroma ovarian tumors and uterine leiomyomas [1]. Ovarian tumors implicated in pseudo-Meigs’s syndrome include struma-ovarii, germ cell tumors, ovarian metastases from gastric or colon cancer and serous or mucinous cystadenomas [2]. Uterine leiomyoma is commonly reported in cases of pseudo-Meigs’ syndrome [3]. Leiomyomas have an incidence of 20–50% in females over the age of 30, but rarely, they are found in association with ascites and hydrothorax [1, 4]. Elevation in CA-125, in addition to the pseudo-Meigs’ syndrome is a rare finding [5–8]. We report a case of a pseudo-Meigs’ syndrome in a 35-year-old female who had markedly elevated CA-125. A literature review was performed, and cases of pseudo-Meigs’ syndrome were reported.

## CASE REPORT

A 35-year-old female presented to the emergency department with complaints of progressive abdominal pain, swelling and weight gain. She denied chest pain, shortness of breath, heat or cold intolerance, palpitations and any motor or sensory deficits. Surgical history was significant for a remote exploratory laparotomy for an abdominal stab wound.

**Figure 1:**
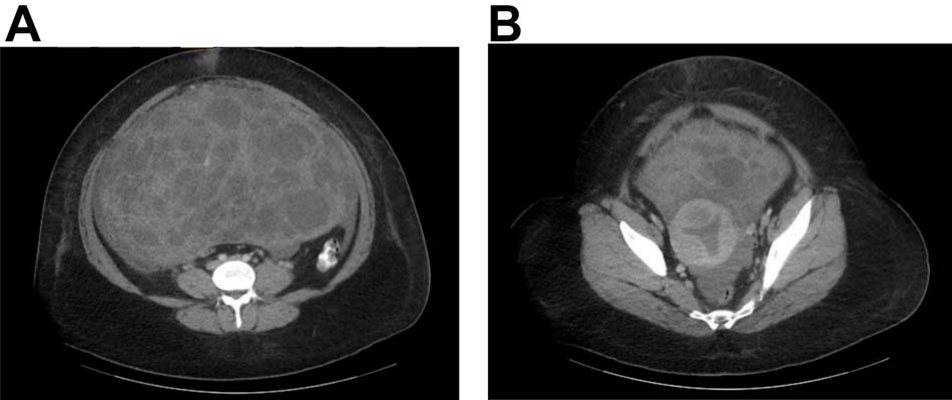
**(A)** CT abdomen and pelvis with contrast identified a large pelviabdominal mass with internal vascularity measuring 29 × 19 cm and significant ascites; the mass contained both cystic and solid components. **(B)** The mass encasing the uterus.

On examination, she was hemodynamically stable. Abdomen was distended and tender to palpation. A fluid shift was present. Computed tomography (CT) abdomen and pelvis demonstrated a 29 × 19-cm pelviabdominal mass with significant ascites. The mass contained both cystic and solid components as well as internal vascularity (Fig. 1A and 1B).

Pregnancy test was negative. She had an elevated CA-125 level of 272.9 ng/ml, suggesting ovarian cancer.

The patient was taken to the operating room for an exploratory laparotomy, excision of intraabdominal mass, hysterectomy and bilateral salpingo-oophorectomy. Upon entry, 2 l of intrabdominal fluid were aspirated and sent to pathology. The mass was identified closely adherent to the anterior abdominal wall, sigmoid colon and two large areas of small bowel. It was dissected off the anterior abdominal wall, sigmoid colon and the two segments of small bowel. The two involved small intestinal segments were resected and anastomosed together.

The tumor, which appeared to originate from the uterine fundus, was completely resected and sent to pathology. A total abdominal hysterectomy and bilateral salpingo-oophorectomy were performed. No intrabdominal metastasis were identified. The abdomen was closed and the patient tolerated the procedure well without complications. Pathology described an aggressive-behaving uterine leiomyoma. Ascitic cytology was negative for malignant cells.

## DISCUSSION

Ascites, pelvic mass and an elevated CA-125 in women carries a grim prognosis, likely an ovarian carcinoma [9, 10]. However, benign etiologies must also be considered [10]. Cases of Meigs’ and pseudo-Meigs’ syndrome with elevations in CA-125 have been reported [10–12].

An elevated CA-125 is not 100% specific for ovarian carcinoma [13]. It can be found in cases of pelvic inflammatory disease, pregnancy, endometriosis, benign ovarian tumors and uterine leiomyomas [14]. In cases of Meigs’, it has been suggested that mesothelial expression of CA-125, rather than expression by the fibroma, is the cause of the elevation [11, 12]. In pseudo-Meigs’ peritoneal inflammation has been implicated as the cause of elevated CA-125 [10].

Clinically, Meigs’ and pseudo-Meigs’ syndrome are indistinguishable. Patients typically present with abdominal distention and a pelvic mass. Some report dyspnea due to pleural effusion [15]. These conditions are distinguished via imaging and pathology. Regardless of the underlying tumor in either Meigs’ or pseudo-Meigs, spontaneous resolution of the pleural effusion and ascites occurs after resection [15]. With this knowledge, surgeons can avoid potential morbidity of unnecessary tests and procedures in the workup and treatment.

The pathophysiology of both Meigs’ and pseudo-Meigs’ syndromes are not entirely known but are thought to be similar [10]. In the formation of ascites, it is suggested that twisting of the tumor around its pedicle results in fluid production [16]. However, this fails to explain ascites in non-pedunculated tumors [10]. Others suggested that ascites could be caused by the mechanical pressure from the tumor on neighboring vessels and lymphatics [1, 17]. This also seems unlikely, as tumors are often freely mobile within the abdomen and would not cause sufficient pressure for fluid transudation [1, 17]. That said, fluid transudation resulting from insufficient venous and lymphatic drainage of a large tumor has also been suggested as a mechanism for ascites [18].

More likely, the formation of ascites and hydrothorax in Meigs’ and pseudo-Meigs’ is multifactorial in nature. It is reported that, intraoperatively, cutting a uterine leiomyoma causes fluid leakage from myometrial cysts within the tumor [10]. Thus, fluid production results from hydropic and subsequent cystic degeneration of the tumor [19]. Pressure within the leiomyoma itself might result in fluid transudation into the abdomen [10]. Also, peritoneal fluid production is also a factor in ascites. Mechanical irritation of the peritoneum from the tumor in addition to pressure from pre-existing ascites causes peritoneal inflammation, which results in further fluid production [20].

The formation of pleural effusion in these syndromes is thought to be a result of transdiaphragmatic transport of the ascitic fluid into the thorax [10]. The mechanism of fluid transport through the diaphragm is likely through intercellular gaps [21, 22]. India ink studies have confirmed the transport of fluid into the pleural space [23].

A literature review using PubMed and Medline was performed, resulted in 21 cases describing pseudo-Meigs’ syndrome [3, 6–8, 10, 13–15, 24–36] (Table 1). Mean age of presentation was 40. Fifteen of 21 cases presented with abdominal distention. Additionally, 8 of 21 cases reported dyspnea. Other reported symptoms were nausea, vomiting, dysuria and urinary frequency. Seventeen articles reported elevated CA-125 (Table 1). Ascites was confirmed in all cases, and hydrothorax was reported in 16 cases. Effusion was demonstrated bilaterally in four patients. Uterine leiomyomas are the most common tumors found in association with pseudo-Meigs’ syndrome [3]. The average tumor size was 17.7 cm. All patients were treated surgically, with no reported cases of tumor recurrence.

**Table 1:** Articles reported pseudo-Meigs’ syndrome cases

Article	Age (Y)	CA 125	Tumor size (cm)	Symptoms	Pleural effusion	Recurrence	FU (M)
Chourmouzi *et al*.2010 [3]	41	436.7 U/ml	16 × 13	Abdominal swelling, discomfort, urinary frequency and incontinence	NS	NS	NS
Ollendorff *et al*. [6]	31	301 U/ml	27 × 18 × 13	Abdominal distention and shortness of breath	Present	NR	8
Domingo *et al*. 1998 [7]	46	317 KAU/l	20	Menorrhagia, fatigue, malaise, pedal edema, abdominal swelling, shortness of breath and respiratory arrest	Present	NS	NS
Dunn *et al*. [8]	46	254 U/ml	30 × 18 × 15	Nausea, vomiting, diarrhea, tachypnea, right pleural effusion and re-accumulation after drainage	Present	NS	NS
Amant et al. 2001 [10]	39	785 kU/l	30 × 30 × 15	Abdominal swelling	Present	NS	NS
Dong *et al*. 2015 [13]	37	920.4 U/ml	20 × 18 × 10	Right lower abdominal dull pain, abdominal distention and nausea	Present	NR	82
Yip *et al*. 2014 [14]	41	939.7 U/ml	12 × 11 × 7.8	Abdominal fullness and prolonged menstrual periods	Absent	NS	NS
Brown *et al*. 1998 [15]	31	83 iu/ml	17 × 11.5 × 8.5	Dyspnea, abdominal swelling, intermittent difficulty in passing urine and 6-kg weight loss	Present	NR	18
Handler *et al*. 1982 [24]	35	NS	9 × 9	Dyspnea, dysmenorrhea and deep dyspareunia	Present	NR	12
Kebapci *et al*. 2002 [25]	38	281 U/ml	10.5 × 10 × 9	Low back pain, distention, weakness and loss of appetite	Present	NS	NS
Migishima *et al*. 2000 [26]	51	820 U/ml	24.3 × 20.5 × 12.3	Abdominal distention and dyspnea	Present	NR	4
Oguma *et al*. 2014 [27]	50	218 U/ml	14 × 8 × 7	Shortness of breath	Present	NS	NS
Ricci *et al*. 2009 [28]	35	231.4 U/ml	15 × 10 × 8.5	Abdominal distention	NS	NR	36
Seo *et al*. 2014 [29]	22	450 kU/l	8.9 × 5.2	Painless abdominal distention	Absent	NR	60
Weinrach *et al*. 2004 [30]	40	734 U/ml	19 × 11 × 10	Abdominal distention and shortness of breath	Present	NR	6
Buckshee *et al*. 1990 [31]	46	NS	20 × 20	Abdominal distention and loss of appetite	Present	NR	NS
Weise *et al*. 2002 [32]	27	1854 U/ml	8 × 7 × 6	Abdominal distention	Present	NR	3
Frank *et al*. 1973 [33]	66	NS	18 × 16 × 13	Dyspnea, fatigue, weakness, palpitations, orthopnea, cough and anorexia	Present	NS	NS
Rush 1976 [34]	47	NS	20	Weakness with exertion, abdominal swelling, constipation, lethargy and blood-streaked stool	Present	NS	NS
Makris *et al*. 2012 [35]	26	93.9 U/ml	11 × 10.2 × 8.3	Pelvic mass	NS	NR	2
Landrum *et al*. 2008 [36]	47	475 U/ml	22 × 20	Upper respiratory infection, anasarca and abdominal distention	Present	NS	NS

Y, years; cm, centimeter; NS, not specified; FU, follow-up; M, months; NR, no recurrence.

## CONCLUSION

Pseudo-Meigs’ syndrome presenting with an elevated CA-125 is a rare clinical entity.

Although it often suggests malignancy, pseudo-Meigs’ syndrome should be considered in the differential diagnosis. Surgery is the mainstay of treatment with subsequent resolution of the ascites and hydrothorax.
